# Association of Chronic Low Back Pain With Personal Space Regulation

**DOI:** 10.3389/fpsyt.2021.719271

**Published:** 2021-12-15

**Authors:** Lin-Man Weng, Bao Wu, Chang-Cheng Chen, Juan Wang, Meng-Si Peng, Zhi-Jie Zhang, Xue-Qiang Wang

**Affiliations:** ^1^Department of Sport Rehabilitation, Shanghai University of Sport, Shanghai, China; ^2^Rehabilitation Therapy Center, Luoyang Orthopedic Hospital of Henan Province, Luoyang, China; ^3^Department of Rehabilitation Medicine, Shanghai Shangti Orthopedic Hospital, Shanghai, China

**Keywords:** interpersonal distance, personal space, low back pain, stop-distance, social behavior

## Abstract

**Background:** While most previous studies regarding patients with chronic low back pain (CLBP) mainly focused on pain, disability, psychological damage, and intervention measures, the effect of CLBP on personal space remains unclear. The study aimed to assess the personal space of patients with CLBP and healthy controls, explored the differences between the two groups, and examined whether pain, dysfunction, anxiety, and depression affected the personal space regulation.

**Methods:** The cross-sectional study recruited 24 patients with CLBP and 24 healthy controls at Shanghai Shangti Orthopedic Hospital and Shanghai University of Sport, Shanghai, China, from December 2018 to January 2019. A stop-distance paradigm was applied to measure the comfortable and uncomfortable distance under four conditions. A self-rating anxiety scale (SAS) and a self-rating depression scale (SDS) were used to examine the anxiety and depression levels of all participants. The pain intensity and dysfunction in the CLBP group were evaluated by the numeric rating scale and Roland-Morris questionnaire (RMDQ), respectively.

**Results:** When approaching another individual or when being approached, the interpersonal distance under all the conditions in the CLBP group significantly differed from that in the healthy control group with larger space distances (*p* < 0.01). Gender had a significant main effect on the regulation of personal space in patients with CLBP (*p* < 0.05). The average pain intensity, scores on RMDQ, SAS, and SDS had a significant positive correlation with the interpersonal distance under the Same or Opposite Gender condition (*p* < 0.05).

**Conclusion:** People with CLBP show an atypical personal space behavior and indeed have a greater interpersonal distance to strangers. The higher the pain intensity, dysfunction, anxiety, and depression, the greater the interpersonal distance in patients with CLBP. In the future, the effect and underlying neural mechanisms of pain and negative emotions on social withdrawal in patients should be examined.

## Introduction

Low back pain (LBP) is defined as pain that occurs between the inferior border of the ribs and the buttock wrinkles, which may be accompanied by lower limb pain or related neurological symptoms (1, 2). Most LBP cases tend to subside within the first 6 weeks (3), while approximately 10% of them will develop into chronic low back pain (CLBP) (4, 5). CLBP, a type of LBP that lasts or fluctuates for more than three months (6), is the most common chronic pain disease that causes clinical, public health, and social problems in the world (7). Normally, 51–80% of adults suffer from CLBP at some point in their lives (8, 9). It also causes a huge economic loss ranging from 12.2 billion to 90.6 billion (6). In addition to pain and impaired function, CLBP limits leisure activities, social activities, and public engagements as well (10). Pain could negatively impact social relationships, such as poor relationships between children and peers (11) and reduced marital satisfaction between partners (12). Furthermore, the associations between CLBP with interpersonal relationships, social withdrawal, and psychological distress, such as depression and loneliness, have been demonstrated (13–15).

Since humans are fully socialized creatures and spend almost 80% of their waking time with other people (16), sociality plays a vital role in their physical and mental health (17). Generally, the lack of sociality is expressed as increased personal space, including increased interpersonal distance, social withdrawal, social isolation, and subjective feelings of loneliness (17, 18). When individuals lack contact with society, they feel social isolation and loneliness and experience social withdrawal. Insufficient social connections and following loneliness might produce pain and depression and result in a higher risk of cardiovascular disease, sleep disorders, suicidal tendencies, and degenerative dementia (17, 19–21). Individuals with social withdrawal likely show poor physical and psychological health. Additionally, a meta-analysis on social relationships and death risk has revealed that the odds ratio of increased mortality due to loneliness is 1.45, which is about twice that caused by obesity (21, 22).

Personal space refers to the perceived comfortable and safe area around individuals. Individuals normally experience discomfort and anxiety if it is intruded (23). During social interaction, they can automatically adjust the distance between themselves and others. Meanwhile, the ability to maintain proper interpersonal distance is conducive to positive and successful social interactions (24). Many studies have explored the adjustment of interpersonal distance among different groups of people. Lough et al. (25) investigated the differences in personal space adjustment between patients with Williams syndrome and typically developing peers. The findings suggested that patients with Williams syndrome more likely invade others' personal space and maintain a shorter interpersonal distance. Furthermore, Rubinsten et al. (26) studied the effect of deficiencies in spatial cognition on personal space regulation and reported that patients with developmental dyscalculia were less efficient in allocating spatial attention and had difficulties in adjusting personal space. Studies have shown that some patients with special diseases lack flexibility in personal space adjustment. It is an important aspect that reflects the social vulnerability profile (25). While a large number of studies regarding on CLBP mainly focused on pain, disability, psychological damage, and intervention measures (27–30), the effect of CLBP on social behavior remains unclear. In other words, it is still unsure whether patients with CLBP can normally adjust their personal space.

The study aimed to assess the personal space of patients with CLBP and healthy controls; explore the differences between the two groups; and examine whether pain, dysfunction, anxiety, and depression affected the personal space regulation in patients with CLBP. It is hypothesized that individuals with CLBP avoid to be too close to strangers and have a greater personal space than healthy controls; pain, dysfunction, anxiety, and depression negatively impact interpersonal distance; and patients with CLBP experience social withdrawal in daily life.

## Methods

### Participants

According to a previous study by Lough et al. (25), the sample size that can be calculated by G^*^Power should have 21 participants in each group (α = 0.05, *d* = 0.8, and power = 80%). In the present study, 24 patients with CLBP and 24 healthy adults were recruited between December 2018 and January 2019 from the Shanghai Shangti Orthopedic Hospital and Shanghai University of Sport, Shanghai, China. The CLBP group (≥18, average 23.83 ± 3.51 years old) and the control group (≥18, average 23.67 ± 1.20 years old) matched in terms of age (23.67 vs. 23.83 years), gender ratio (45.83% vs. 33.33% male), height (166.38 vs. 167.06 cm), body weight (63.23 vs. 62.23 kg), and body mass index (BMI, 22.73 vs. 22.21 kg/m^2^) (Table 1). All the participants met the inclusion and exclusion criteria, and they were instructed to abstain from alcohol or drugs for 24 h before the experiment. The healthy controls were included on the basis of the following criteria: (1) age over 18 years, (2) no chronic or acute pain in the body, and (3) able to communicate and understand the instructions well. They were excluded in accordance with the following criteria: (1) a history of neurologic or psychiatric disorders, (2) a history of substance use and abuse, (3) currently taking anti-depressants or analgesics, and (4) pregnant or lactating. The subjects with CLBP were included on the basis of the following criteria: (1) age over 18 years, (2) pain in the back area from below the costal margin to the gluteal fold and lasting at least 12 weeks with an average pain intensity of 3 or greater on an 11-point numerical rating scale in the previous week, (3) no other pain or pain unrelated to the lower back and no dysfunction in the body, and (4) able to communicate and understand the instructions well. The subjects with CLBP were excluded in accordance with the following criteria: (1) low back pain caused by intervertebral disk diseases, paravertebral infection or tumor, spinal diseases or injury, and visceral diseases, (2) a history of neurologic or psychiatric disorders, (3) a history of substance use and abuse, (4) currently taking anti-depressants or analgesics; and (5) pregnant or lactating.

**Table 1:** Baseline characteristic of participants.

**Characteristic**	**Low Back Pain Group (*n* = 24)**	**Control Group** **(*n* = 24)**	***P* value**
Males/females	8/16	11/13	0.376
Age, years	23.83 ± 3.51	23.67 ± 1.20	0.827
Height, cm	167.06 ± 9.18	166.38 ± 7.62	0.782
Body weight, kg	62.23 ± 10.23	63.23 ± 10.69	0.741
BMI, kg/m^2^	22.21 ± 2.58	22.73 ± 2.70	0.497
SAS score	45.25 ± 9.60	35.00 ± 5.83	<0.001^*^
SDS score	44.50 ± 12.17	37.13 ± 7.13	0.014
NRS1	5.41 ± 1.64		
NRS2	3.29 ± 1.16		
RMDQ	6.13 ± 3.97		

* *p < 0.05*.

*>Data are presented as number or as mean ± SD*.

*BMI, body mass index; SAS, self-rating anxiety scale; SDS, self-rating depression scale; NRS1, the maximum pain intensity during the subjects experiencing low back pain; NRS2, the average pain intensity during the subjects experiencing low back pain; RMDQ, Roland Morris disability questionnaire*.

The Ethics Committee of Shanghai University of Sport approved this study. All the subjects voluntarily participated and signed their written informed consent forms.

### Assessment

Before participating in the study, all the subjects completed a questionnaire on the following details: basic information, CLBP evaluation, and psychological evaluation (healthy subjects only completed basic information and psychological evaluation).

In CLBP evaluation, a numeric rating scale (NRS, 0–10, where 0 indicated “no pain at all,” and 10 denoted “the worst imaginable pain”) was used to assess the maximum and average pain intensity (NRS1 and NRS2) of the patients who experienced CLBP in the past 3 days. It is a valid, reliable, has good test-rest reliability (intraclass correlation coefficient, ICC = 0.991), excellent responsiveness (area under the curve, AUC = 0.880), and is often preferred tool to measure the severity of pain (31, 32). The dysfunction related to CLBP was examined with the Roland-Morris questionnaire (RMDQ), which is frequently utilized to measure the specific functional status of the back (33). RMDQ includes 24 items scored as 0 or 1, where 1 indicates that an item is applicable to the subjects, and 0 denotes that an item is not applicable. The total score range is 0 (no disability) to 24 (the highest possible disability). It has good test-retest reliability (ICC = 0.855), responsiveness (AUC = 0.868), internal consistency, construct validity, and face and content validity (32, 34).

In psychological evaluation, all the subjects were examined with a self-rating anxiety scale (SAS) and a self-rating depression scale (SDS). These scales are used to quantify the anxiety and depression level of the subjects. Each scale has 20 items with a score of 1–4 (1 = never, 2 = often, 3 = sometimes, and 4 = always). The normalized score is equal to the total score (ranging from 20 to 80) multiplied by 1.25 (SAS: 20–44 = normal range, 45–59 = mild anxiety, 60–74 = moderate anxiety, 75–80 = severe anxiety; SDS: 20–49 = normal range, 50–59 = mild depression, 60–69 = moderate depression, 70–80 = severe depression) (35). Cronbach's coefficients of the Chinese version of the SAS and SDS used in this study are 0.931 and 0.784, respectively (36). The SAS is significantly correlated with the Taylor Manifest Anxiety Scale (*r* = 0.30), and the correlation between the SDS and the Beck Depression Inventory is 0.68 (37). Both scales have good internal consistency, reliability, and validity (36). The SAS and the SDS yield convincing results of anxiety and depression assessment, and they are widely applied to different areas, such as rehabilitation, psychiatry, and oncology (38, 39).

### Procedure

In social interaction, people can automatically regulate interpersonal distance. With high validity and reliability, a stop-distance paradigm is frequently applied to measure the preferred interpersonal distance under various conditions (25, 40). At the beginning of a task, a subject and the experimenter faced each other at a distance of 3 m. Four conditions were set: two of them involved completing a task with an unfamiliar person of the same gender (the experimenter was the same gender as the subject), and the two other conditions were undertaken with an unfamiliar person of the opposite gender (the experimenter was the opposite gender as the subject).

**Figure 1:**
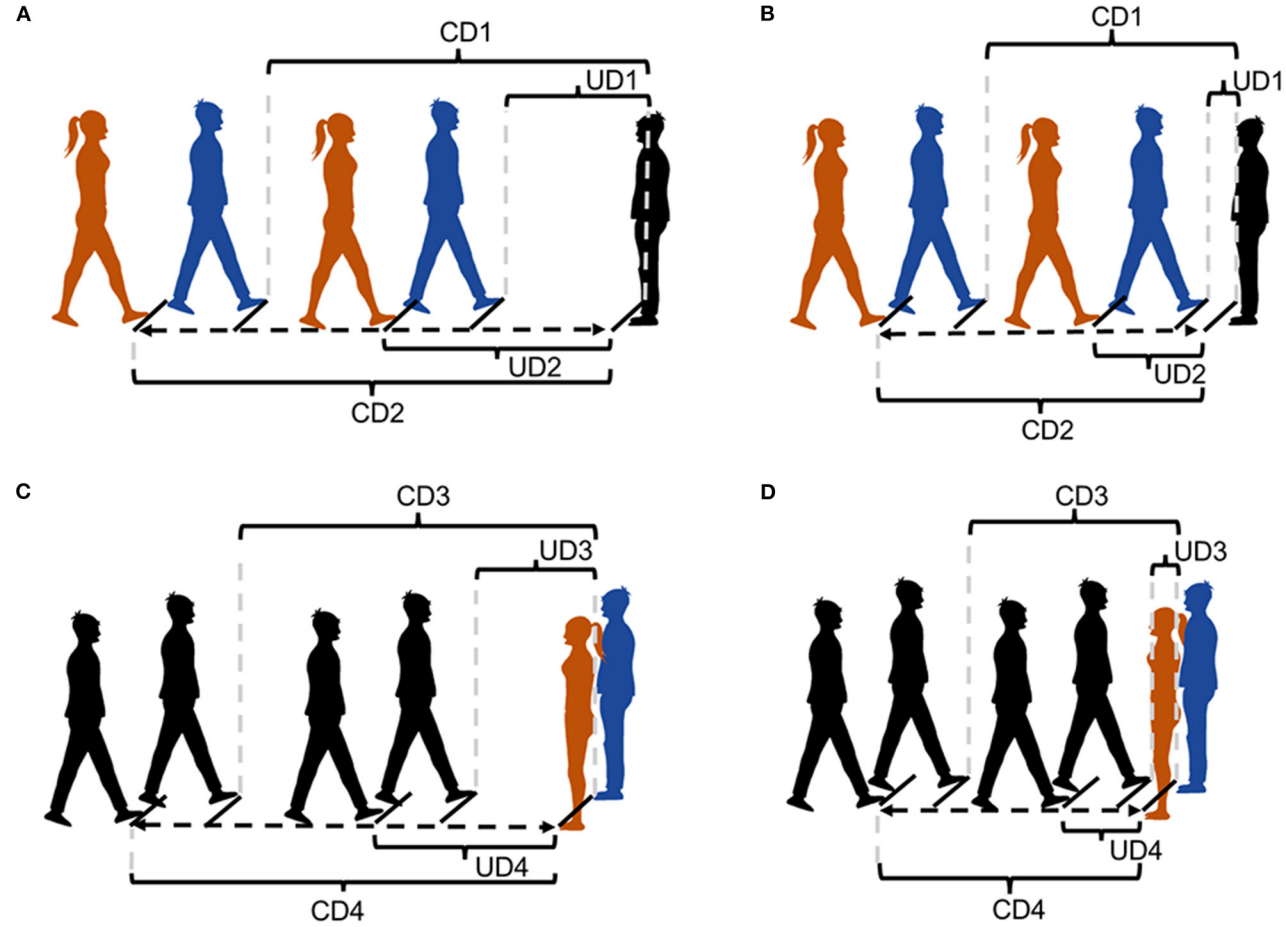
Stop-distance paradigm. **(A)** The interpersonal distance between participant with CLBP and experimenter under the condition of being approached. **(B)** The interpersonal distance between health control and experimenter under the condition of being approached. **(C)** The interpersonal distance between participant with CLBP and experimenter under the condition of active approach. **(D)** The interpersonal distance between health control and experimenter under the condition of active approach. Black figures: the participant. Blue figures: the experimenters (the same gender as the participant). Red figures: the experimenters (the opposite gender as the participant). CD1: The comfortable distance between the participant and the experimenter (same gender) under the condition of being approached. CD2: The comfortable distance between the participant and the experimenter (opposite gender) under the condition of being approached. UD1: The uncomfortable distance between the participant and the experimenter (same gender) under the condition of being approached. UD2: The uncomfortable distance between the participant and the experimenter (opposite gender) under the condition of being approached. CD3: The comfortable distance between the participant and the experimenter (same gender) under the condition of active approach. CD4: The comfortable distance between the participant and the experimenter (opposite gender) under the condition of active approach. UD3: The uncomfortable distance between the participant and the experimenter (same gender) under the condition of active approach. UD4: The uncomfortable distance between the participant and the experimenter (opposite gender) under the condition of active approach.

Under the first condition, the unfamiliar experimenter (same gender) slowly walked toward the subject until he or she reached the distance, where the subject would normally maintain a stranger; this distance was denoted as the comfortable distance (CD1; Figure 1). Thereafter, the unfamiliar experimenter (same gender) continued to move toward the subject and stopped at the distance, where the subject felt uncomfortable; this distance was indicated as the uncomfortable distance (UD1; Figure 1). Afterward, the procedure was repeated, but the subject walked toward the unfamiliar experimenter (same gender) and stopped again at comfortable (CD3; Figure 1) and uncomfortable distances (UD3; Figure 1). The task was repeated, but an unfamiliar experimenter of the opposite gender was involved this time (comfortable distance: CD2 and CD4; uncomfortable distances: UD2 and UD4; Figure 1). Toe-to-toe distances were tested three times under each condition and recorded using a digital laser measurer (Bosch GLM 30C). The average of being approached by the same-gender experimenter and the opposite-gender experimenter and approaching them was taken as the outcome measure (41, 42). Different experimenters were randomly assigned under each condition to avoid familiarity effects (17).

### Statistical Analyses

Differences in demographical and clinical data between the two groups were explored with a chi-square test, an independent sample *t*-test, and a Wilcoxon rank-sum test. We performed a normality test on all data and normal transformation when necessary. The difference in the interpersonal distance under various conditions between the CLBP and control groups was analyzed through an independent sample *t*-test. Two-way ANOVA was performed to examine the significant effect of the Approach Direction (being approached or approaching) and Gender (same or opposite gender) on interpersonal distance based on adjusted analysis. Age, body weight, height, and BMI were included in adjusted analysis as baseline covariates. Further associations between interpersonal distance and different clinical indicators (SAS, SDS, NRS1, NRS2, and RMDQ) were tested by using the Pearson correlation test with Benjamini-Hochberg adjustment for multiple testing. Adjusted *p* < 0.05 was considered as statistical significance.

## Results

### Stop-Distance Paradigm

#### Being Approached by an Unfamiliar Experimenter

**Figure 2:**
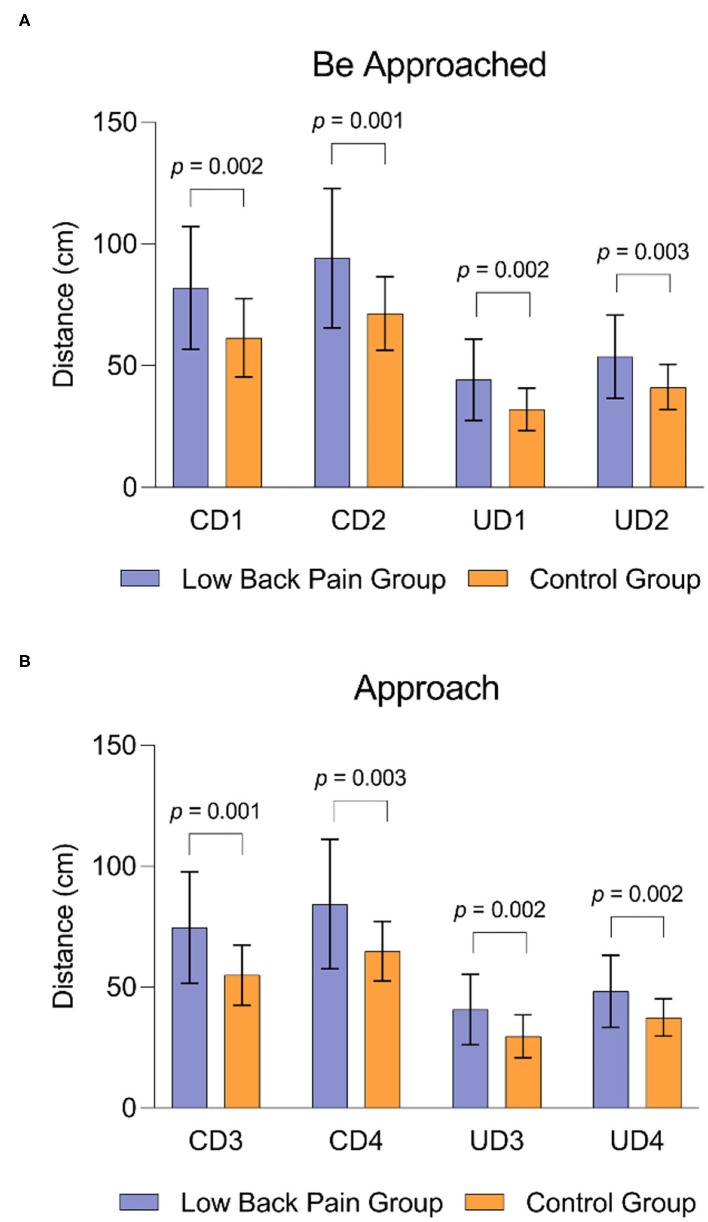
The difference in interpersonal distance across the CLBP and control group. **(A)** The difference in interpersonal distance across the CLBP and control group under the condition of being approached. **(B)** The difference in interpersonal distance across the CLBP and control group under the condition of active approach.

Under the Same Gender condition, the average CD1 between the subjects and an unfamiliar experimenter was 81.97 ± 25.25 cm in the CLBP group and 61.49 ± 16.11 cm in the control group, and they had average UD1 of 44.15 ± 16.77 and 32.01 ± 8.75 cm, respectively (Figures 1A,B). Under the Opposite Gender condition, the average CD2 was 94.17 ± 28.61 cm in the CLBP group and 71.44 ± 15.17 cm in the control group, and they had average UD2 of 53.69 ± 20.40 and 41.21 ± 9.29 cm, respectively (Figures 1A,B). When the subject was approached by the experimenter, the distance under all the conditions in the CLBP group significantly differed from that in the healthy control group with larger space distances (CD1: *t* = 3.350, *p* = 0.002; UD1: *t* = 3.251, *p* = 0.002; CD2: *t* = 3.496, *p* = 0.001; UD2: *t* = 3.208, *p* = 0.003; Figure 2A).

#### Approaching an Unfamiliar Experimenter

Under the Same Gender condition, the average CD3 between the subjects and an unfamiliar experimenter was 74.63 ± 23.09 cm in the CLBP group and 55.02 ± 12.40 cm in the control group, and they had average UD3 of 40.75 ± 14.51 and 29.68 ± 8.91 cm, respectively (Figures 1C,D). Under the Opposite Gender condition, the average CD4 was 84.34 ± 26.84 cm in the CLBP group and 64.86 ± 12.35 cm in the control group, and they had average UD4 of 48.31 ± 14.91 and 37.47 ± 7.71 cm, respectively (Figures 1C,D). When the subject approached the experimenter, the distance under all the conditions in the CLBP group significantly differed from that in the healthy control group with larger space distances (CD3: *t* = 3.734, *p* = 0.001; UD3: *t* = 3.258, *p* = 0.002; CD4: *t* = 3.229, *p* = 0.003; UD4: *t* = 3.216, *p* = 0.002; Figure 2B).

### Factors Affecting Personal Space

#### Approach Direction or Gender

##### Comfortable Distance

The Approach Direction significantly affected the control group but not the CLBP group [*F*_(1,93)_ = 5.187, *p* = 0.025; $\eta_{p}^{2}$ = 0.053; Table 2]. The result indicated that the subjects maintained a larger interpersonal distance when they were approached by the experimenter compared with that when they approached actively the experimenter. Gender also had a significant main effect [*F*_(1,93)_ = 11.943, *p* = 0.001; $\eta_{p}^{2}$ = 0.114], as participants showed a greater distance with the experimenter of the opposite gender compared with that of the experimenter of the same gender in the healthy control group. However, no significant interaction between Approach Direction and Gender was observed [*F*_(1,88)_ < 0.001, *p* = 0.991; $\eta_{p}^{2}$ < 0.001]. In addition, the Same- and Opposite-Gender subgroups significantly differed in terms of the comfortable distance when they were being approached or actively approaching the experimenter (Being Approached: *t* = −2.203, *p* = 0.033; Approaching: *t* = −2.756, *p* = 0.008). No significant difference in the distance of the Being Approach subgroup and the Approaching subgroup under the Same or Opposite Gender condition (Same Gender: *t* = 1.559, *p* = 0.126; Opposite Gender: *t* = 1.646, *p* = 0.167).

**Table 2:** The effect of the Approach Direction (being approached or approaching) and Gender (same or opposite gender) on interpersonal distance.

	**Comfortable Distance**		**Uncomfortable Distance**	
	**Low Back Pain Group**	**Control Group**	**Low Back Pain Group**	**Control Group**
Be approached or approach	2.638	5.187^*^	1.858	2.954
Same gender or opposite gender	4.293^*^	11.943^*^	7.051^*^	23.171^*^
Direction of approach * Gender	0.035	<0.001	0.046	0.158

* *p < 0.05*.

*Data are presented as F*.

In the CLBP group, Gender showed a significant main effect [*F*_(1,93)_ = 4.293, *p* = 0.041; $\eta_{p}^{2}$ = 0.044]. However, the Approach Direction had no significant main effect [*F*_(1,93)_ = 2.638, *p* = 0.108; $\eta_{p}^{2}$ = 0.028], and no significant interaction was observed between the two factors [*F*_(1,88)_ = 0.003, *p* = 0.960; $\eta_{p}^{2}$ < 0.001]. Statistical analysis revealed no significant difference between the Same- and Opposite-Gender subgroups or Being Approached and Approaching subgroups (Being Approached: *t* = −1.609, *p* = 0.114; Approaching: *t* = −1.375, *p* = 0.176; Same Gender: *t* = 1.068, *p* = 0.291; Opposite Gender: *t* = 1.280, *p* = 0.228).

##### Uncomfortable Distance

Gender had a significant main effect in the control and CLBP groups [control group: *F*_(1,93)_ = 23.171, *p* < 0.001; $\eta_{p}^{2}$ = 0.199; CLBP group: *F*_(1,93)_ = 7.051, *p* = 0.009; $\eta_{p}^{2}$ = 0.070; Table 2]. The result showed that the subject had a closer distance with the experimenter of the same gender than that of the opposite gender. However, Approach Direction had no significant main effect [control group: *F*_(1,93)_ = 2.954, *p* = 0.089; $\eta_{p}^{2}$ = 0.031; CLBP group: *F*_(1,93)_ = 1.858, *p* = 0.176; $\eta_{p}^{2}$ = 0.020], and the two factors also had no significant interaction [control group: *F*_(1,88)_ = 0.019, *p* = 0.892; $\eta_{p}^{2}$ < 0.001; CLBP group: *F*_(1,88)_ = 0.002, *p* = 0.962; $\eta_{p}^{2}$ < 0.001]. In the healthy control group, the Same- and Opposite-Gender subgroups significantly differed when they were being approached or actively approaching the experimenter (approached: *t* = −3.531, *p* = 0.001; approaching: *t* = −3.239, *p* = 0.002). The distance maintained by the Opposite-Gender subgroup was constantly larger than that maintained by the Same-Gender subgroup. The Being Approach subgroup and the Approaching subgroup did not significantly differ in terms of uncomfortable distance under the Same or Opposite Gender conditions (Same Gender: *t* = 0.914, *p* = 0.366; Opposite Gender: *t* = 1.517, *p* = 0.136). In the CLBP group, no significant difference was observed between the Same- and Opposite-Gender subgroups or the Being Approached and Approaching subgroups (Being approached: *t* = −2.117, *p* = 0.040; Approaching: *t* = −1.936, *p* = 0.059; Same Gender: *t* = 0.764, *p* = 0.449; Opposite Gender: *t* = 1.188, *p* = 0.241).

#### Clinical Variables

**Figure 3:**
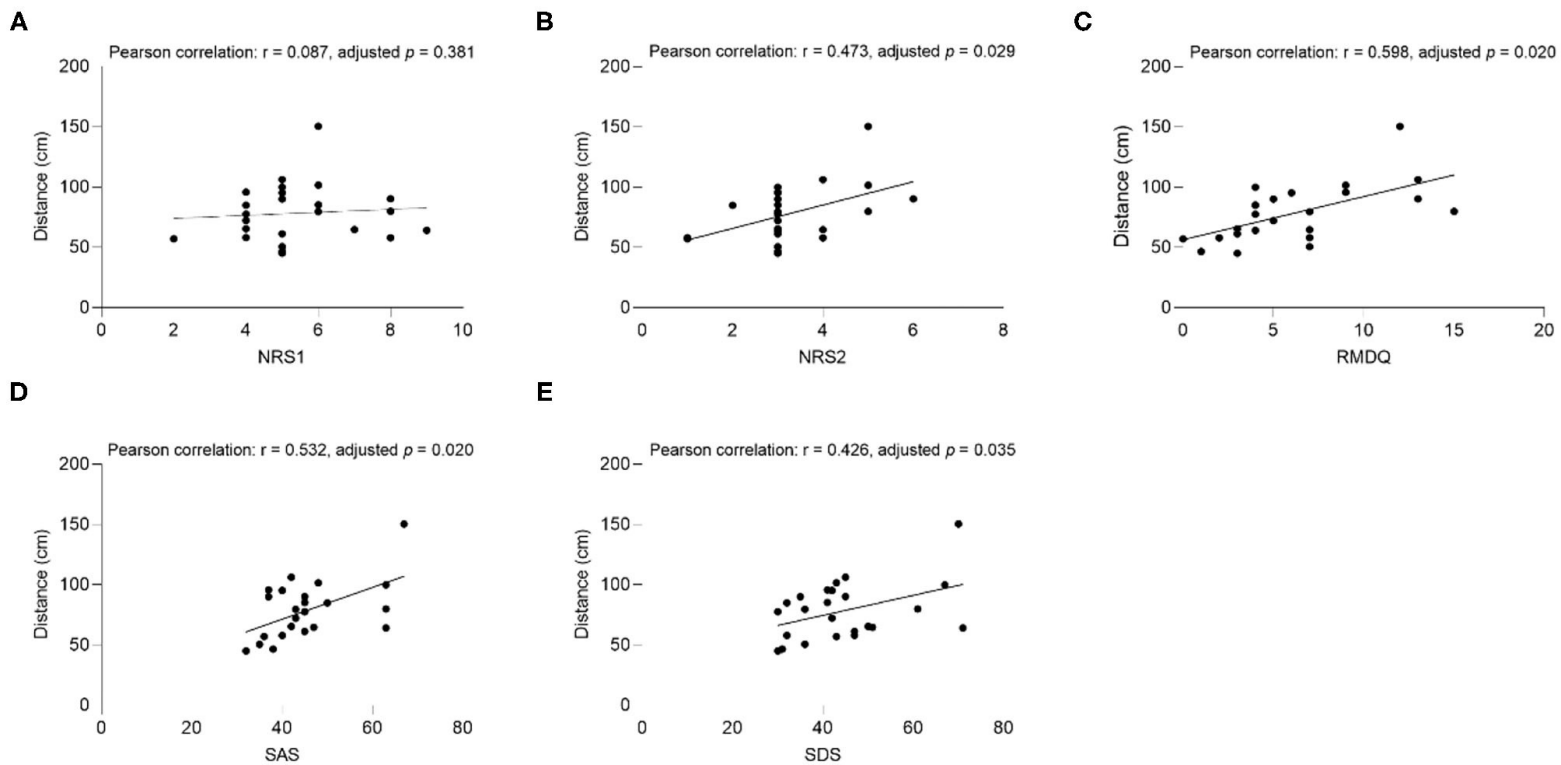
The correlation between comfortable distance and **(A)** NRS1, **(B)** NRS2, **(C)** RMDQ, **(D)** SAS, **(E)** SDS in CLBP group under the Same Gender condition. NRS1, the maximum pain intensity during the subjects experiencing low back pain; NRS2, the average pain intensity during the subjects experiencing low back pain; RMDQ, Roland-Morris questionnaire; SAS, self-rating anxiety scale; SDS, self-rating depression scale.

**Figure 4:**
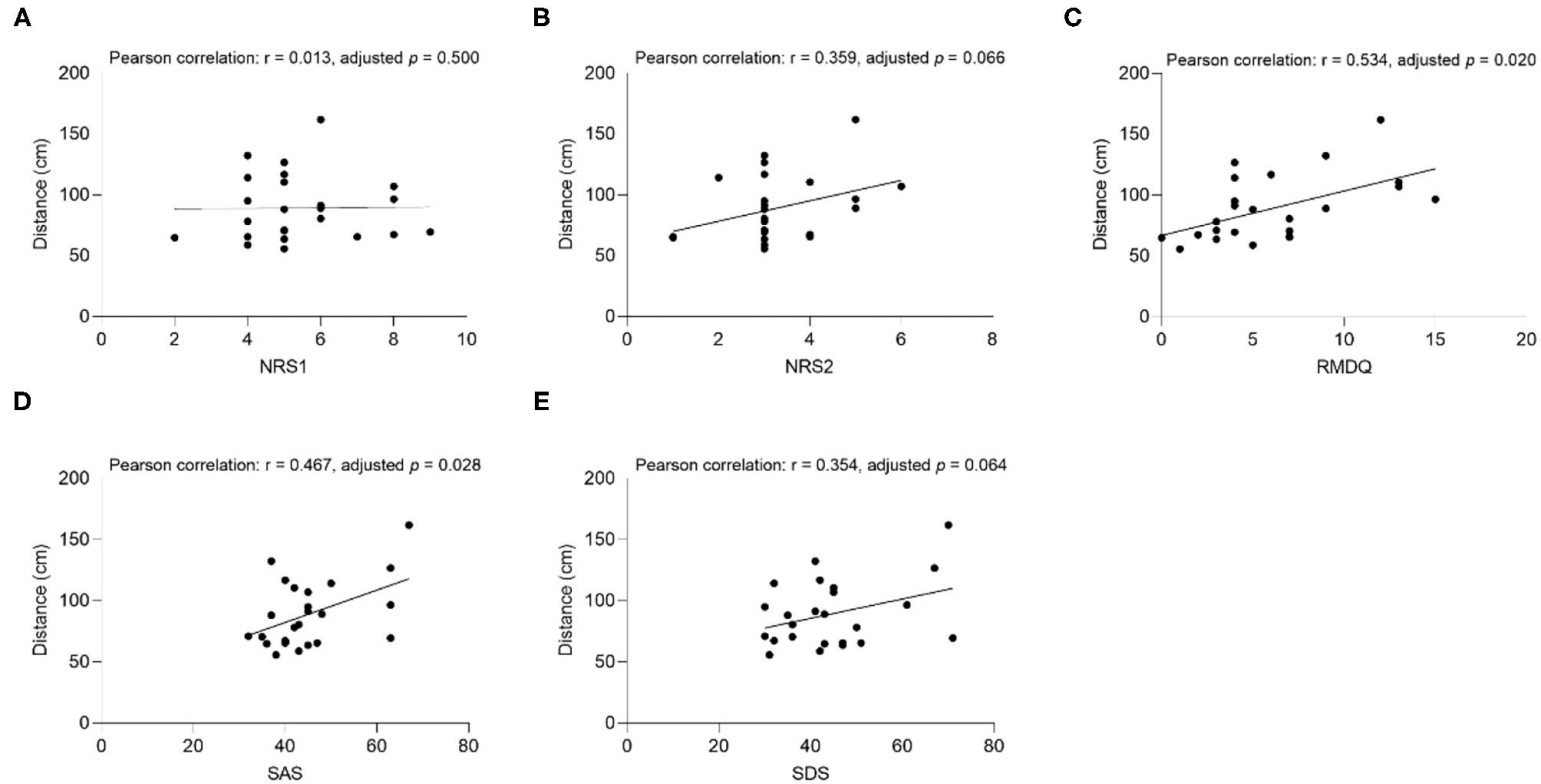
The correlation between comfortable distance and **(A)** NRS1, **(B)** NRS2, **(C)** RMDQ, **(D)** SAS, **(E)** SDS in CLBP group under the Opposite Gender condition. NRS1, the maximum pain intensity during the subjects experiencing low back pain; NRS2, the average pain intensity during the subjects experiencing low back pain; RMDQ, Roland-Morris questionnaire; SAS, self-rating anxiety scale; SDS, self-rating depression scale.

**Figure 5:**
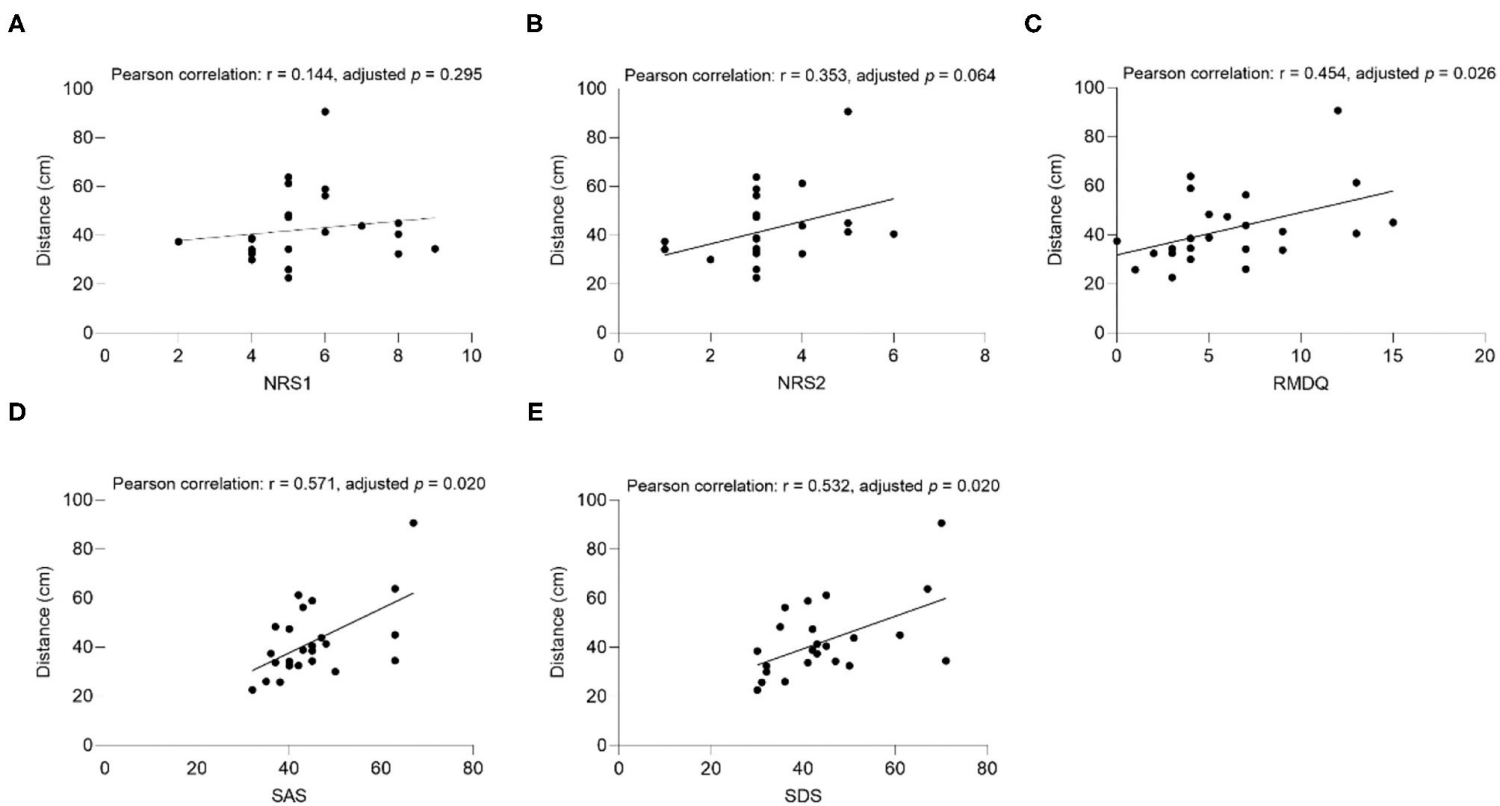
The correlation between uncomfortable distance and **(A)** NRS1, **(B)** NRS2, **(C)** RMDQ, **(D)** SAS, **(E)** SDS in CLBP group under the Same Gender condition. NRS1, the maximum pain intensity during the subjects experiencing low back pain; NRS2, the average pain intensity during the subjects experiencing low back pain; RMDQ, Roland-Morris questionnaire; SAS, self-rating anxiety scale; SDS, self-rating depression scale.

**Figure 6:**
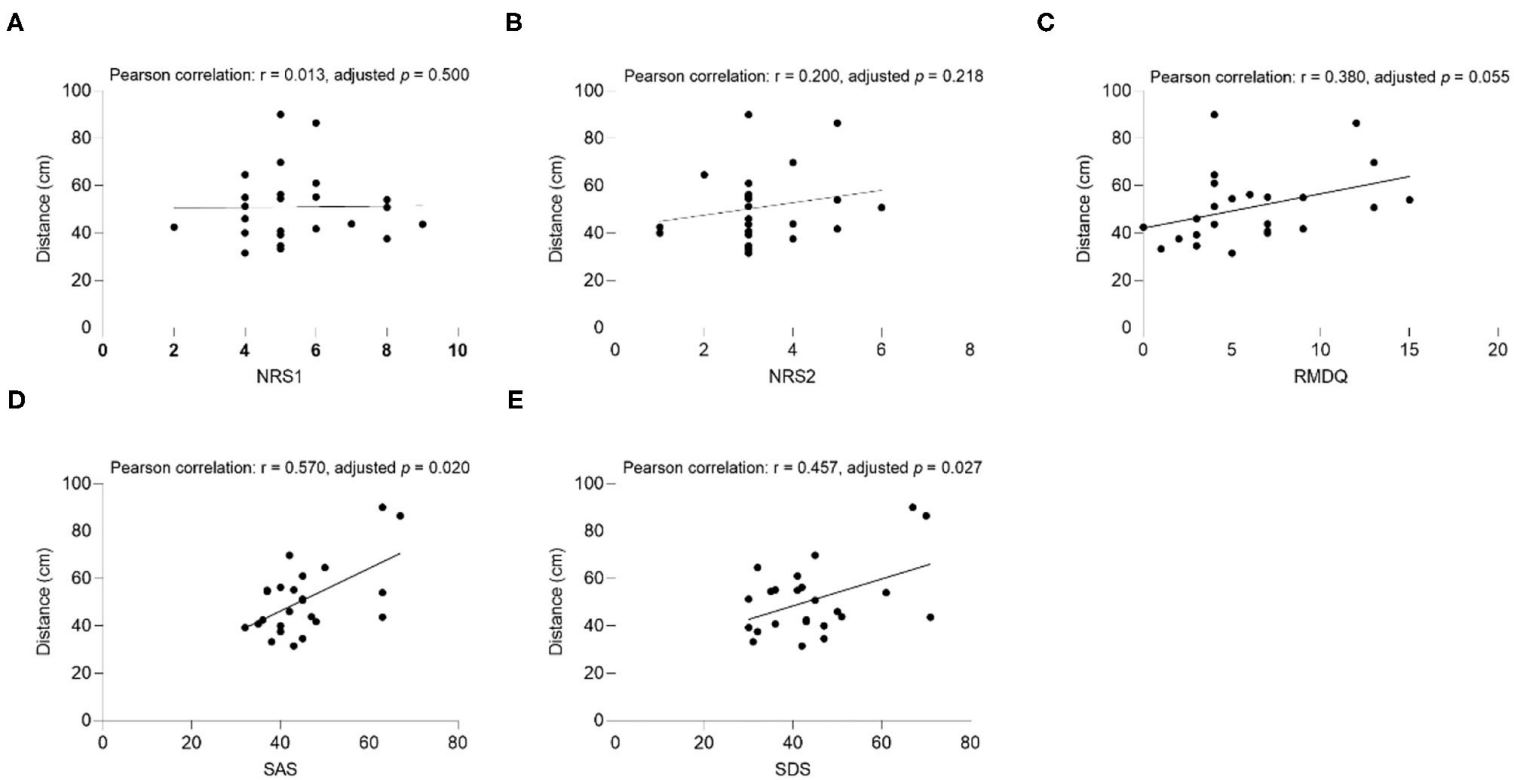
The correlation between uncomfortable distance and **(A)** NRS1, **(B)** NRS2, **(C)** RMDQ, **(D)** SAS, **(E)** SDS in CLBP group under the Opposite Gender condition. NRS, numerical rating scale; RMDQ, Roland-Morris questionnaire; SAS, self-rating anxiety scale; SDS: self-rating depression scale.

The results of the correlation analysis between clinical factors (SAS, SDS, NRS1, NRS2, and RMDQ) and interpersonal distance are shown in Figures 3–**6**.

##### Comfortable Distance

In the CLBP group, NRS2 (Pearson correlation: *r* = 0.473, adjusted *p* = 0.029), RMDQ (Pearson correlation: *r* = 0.598, adjusted *p* = 0.020), SAS (Pearson correlation: *r* = 0.532, adjusted *p* = 0.020), SDS (Pearson correlation: *r* = 0.426, adjusted *p* = 0.035), and comfortable distance had significant positive associations when the subjects were the same gender as the experimenter (Figure 3). RMDQ (Pearson correlation: *r* = 0.534, adjusted *p* = 0.020), SAS (Pearson correlation: *r* = 0.467, *p* = 0.028), and distance had significant positive associations when the gender of the subjects and the experimenter were not the same (Figure 4). In the healthy control group, no clinical factors were significantly related to the interpersonal distance under various conditions.

##### Uncomfortable Distance

The results showed that the uncomfortable distance had a significant positive correlation with RMDQ (Pearson correlation: *r* = 0.454, adjusted *p* = 0.026), SAS (Pearson correlation: *r* = 0.571, adjusted *p* = 0.020), and SDS (Pearson correlation: *r* = 0.532, adjusted *p* = 0.020) under the Same Gender condition of the CLBP group (Figure 5). The correlation of SAS (Pearson correlation: *r* = 0.570, adjusted *p* = 0.020), and SDS (Pearson correlation: *r* = 0.457, *p* = 0.027) under the Opposite Gender condition was significantly positive (Figure 6). In the healthy control group, all the clinical factors and the interpersonal distance were not significantly correlated under different conditions.

## Discussion

The results revealed that the individuals with CLBP generally showed a greater personal space than the healthy controls. When approaching a stranger or when being approached, the patients kept a greater interpersonal distance from the stranger of the same gender or opposite gender. Meanwhile, the interpersonal distance between patients and the stranger of the same gender was shorter than that between patients and the stranger of the opposite gender. Furthermore, the higher the pain intensity, dysfunction, anxiety, and depression, the greater the interpersonal distance in patients with CLBP.

### The Effect of CLBP on the Personal Space

The individuals with CLBP showed a significantly greater personal space than the healthy controls, which indicated that patients with CLBP consciously avoided further contact with others and expressed greater social withdrawal. This phenomenon has also been reported in patients suffering from other pain. For example, Lutzman et al. (43) found that single elderly people with physical pain felt a greater loneliness and showed a lower social integration than those without pain. Furthermore, Stout et al. (44) showed that women who experienced more intense and frequent pain during intercourse could suffer from more loneliness and depression. In the task of Cyberball social exclusion, participants who received endotoxin experienced worse social disconnection than participants who received the same volume of saline (45). These studies pointed out the relationship between physical pain and social withdrawal. One possible explanation for this phenomenon is that physical pain restricts individuals from participating in social activities and maintaining interpersonal relationships. Another one is that patients try to hide their pain by minimizing contact with the outside world, thereby producing social withdrawal and loneliness. To explain further, CLBP often negatively affects self-perception, which is patients are ashamed of difficulties in performing daily living activities and they are often misunderstood by others due to the absence of obvious signs or symptoms. Therefore, they cannot reasonably manage their social interactions (46). Furthermore, from the perspective of neural mechanisms, this phenomenon is probably due to the dependence of physical pain and social pain on a shared neural circuit (47–49). As an important area responsible for processing unpleasant and negative emotions related to social events or physical pain (48), the pregenual anterior cingulate cortex (pACC), for instance, is responsible for accepting physical pain regulation and gives a change in social pain. Similarly, the activation of the dorsal anterior cingulate cortex (dACC) decreases with the pain subsides, thereby reducing social pain (49).

In the stop-distance task, the gender played a vital role in personal space regulation in both groups, which suggested that the distance between individuals of the same gender was shorter than that between individuals of the opposite gender. The gender difference could be explained by neural mechanisms. The activation of the amygdala in children when encountering opposite-gender faces is significantly stronger than when interacting with same-gender faces (50). The amygdala is activated during close contact with others, thereby maintaining the minimum distance between people (40). This early development of gender differences has potential long-term effects that is individuals prefer to choose the same-gender playmates rather than the opposite-gender (50), which is a possible reason for participants showed shorter interpersonal distances when facing the same gender in this study.

### Other Factors for Personal Space

Pain intensity, dysfunction, anxiety, and depression negatively affected the personal space regulation of patients with CLBP, which means the serious health problems are associated with increased interpersonal distance. From the perspective of pain intensity, individuals with CLBP who are afraid of pain likely develop catastrophic pain cognitions to avoid activities that might irritate more serious pain and harm. The presence of the avoidance behaviors subsequently leads to increased disability and depression, which in turn increases pain intensity (51). Pain and disability limit social participation in patients with CLBP. In terms of anxiety and depression, previous studies have reported that increased negative emotions can be accompanied by loneliness and social separation (17). People with high anxiety usually adopt an avoidant coping strategy in social interactions (52). Similarly, depressive symptoms could reduce people's social interaction time, and people with depressive symptoms tend to stay away from social networks and be isolated (53). Overall, individuals with anxiety, depression, or anxiety-depressive comorbidities show severe social dysfunction (54). As can be seen, the anxiety and depression symptoms in patients with CLBP may further reduce social interaction. There are some possible reasons why anxiety and depression negatively affect personal space. The first one is that the signal transduction of the brain reward system, which is closely related to the amygdala, in patients with anxiety or depression is impaired. It makes patients think that the rewards from social interaction are fewer and express social avoidance and withdrawal (54, 55). Another reason could be that the basolateral amygdala complex (BLA) exhibits hyperactivity in anxiety disorders and decreases social interaction by activating the BLA- medial prefrontal cortex projection (56).

As can be seen, the amygdala plays a key role in emotional disorders (e.g., depression, anxiety) and social behaviors. Additionally, considering its bilateral activation is correlated with the perceived pain intensity (57), the amygdala may be also a key brain area that regulates the social interaction in patients with CLBP and can be used to interpret the appearance of social withdrawal. It has been demonstrated that patients with CLBP have functional and structural changes in various brain regions, including the amygdala that belongs to the emotion-related circuitry (58–60). Resting-state functional magnetic resonance imaging (fMRI) has shown the increased activation of the amygdala in patients with CLBP (58, 61). Rodriguez-Raecke et al. (62) reported that the activation of the amygdala in patients with CLPB or depression increases during pain stimulation, and depression and pain seem to reinforce each other clinically. The pain and negative emotions in patients with CLBP could be connected through the amygdala, which is directly related to personal space regulation. Consequently, when CLBP or chronic pain occurs, the plasticity of the central nervous system changes, and the amygdala, a part of the cognitive-affective system of the “pain matrix,” is activated. Patients experience emotional disorders and atypical social behavior, which appears a larger personal space than that in healthy people.

Interestingly, the results showed that there is no association between maximum pain intensity during CLBP and personal space. One possible reason is that the maximum pain intensity during CLBP does not have a continuous and stable effect, which might indicate a limited effect of pain on the regulation of personal space in social communication. In the future, studies should explore whether immediate painful stimulation affects the size of personal space.

### Limitations

Few studies have explored the effect of CLBP on personal space. This study provided new insight into the personal space management for patients with CLBP, which is practically significant. However, the limitations in the study should be considered. First, the sample size was relatively small. In future experiments, more subjects should be recruited to improve the credibility of experimental results. From the perspective of participant recruitment, a wider age range and more occupational types should be considered to more comprehensively show the results. Second, a stop-distance paradigm as an artificial task may fail to completely and accurately reflect social behavior in real life. As such, tasks that can more validly reflect the personal space during real social interactions should be established. Third, this study only investigated the changes in interpersonal distance between patients with CLBP and strangers. Further studies should examine whether the interpersonal distance between patients and familiar people will be different from healthy controls. Fourth, other factors that may affect social behavior and personal space, such as employment status, socioeconomic status, were not collected in the study. These factors could be considered and included in the next step of research.

### Clinical Implications and Future Research

Our results revealed that the association of CLBP with social withdrawal is worthy to be focused on. This study offered a novel method for assessing the psychosocial status in patients with CLBP. Pain and disability are the most prominent symptoms in CLBP, which makes most patients seek medical attention. Psychosocial problems, which are often overlooked, play an important role in physical and mental health. As such, the psychosocial problems in patients with CLBP should be explored. Medical staff should consider potential social withdrawal and reduced social interactions. They should also formulate the corresponding psychosocial interventions to help patients smoothly reintegrate into society. Additionally, the effect and underlying neural mechanisms of pain and negative emotions on social withdrawal in patients should be examined. In the future, a larger sample size, more realistic social tasks, and more task models (such as those determining whether the interpersonal distance between patients with CLBP and familiar people will be different from healthy controls) will be needed to explore the effect of CLBP on psychosocial status.

## Conclusion

People with CLBP show an atypical personal space behavior, indeed have a greater interpersonal distance to strangers and reduced social interactions. The increased anxiety, depression, average pain intensity, and dysfunction in CLBP are related to the greater personal space and more severe social withdrawal. The changes of personal space are one of the manifestations of social behavior. CLBP reduces the connection and interaction between individuals and society and produces social withdrawal and loneliness. In addition to alleviating pain and dysfunction in clinic, social and psychological problems in patients with CLBP during recovery should be explored to accurately identify the difficulties that need improvement. Further research can enhance our understanding of psychosocial changes in patients with CLBP and provide the evidence for the implementation of psychological rehabilitation.

## Data Availability Statement

The original contributions presented in the study are included in the article, further inquiries can be directed to the corresponding authors.

## Ethics Statement

The studies involving human participants were reviewed and approved by Ethics Committee of Shanghai University of Sport. The patients/participants provided their written informed consent to participate in this study.

## Author Contributions

L-MW, BW, and X-QW conceived and designed the study. L-MW, BW, C-CC, JW, and M-SP collected the data. L-MW and BW analyzed and interpreted the data. L-MW drafted the manuscript. BW, Z-JZ, and X-QW revised the manuscript for important intellectual content. All authors discussed the results, commented on the manuscript, and approved the final manuscript.

## Funding

This work was supported by Fok Ying-Tong Education Foundation of China (161092), Scientific and Technological Research Program of the Shanghai Science and Technology Committee (19080503100), Shanghai Key Lab of Human Performance (Shanghai University of Sport) (11DZ2261100), Science and Technology Commission of Shanghai Municipality (21S31902400), and Project of Science Research of Traditional Chinese Medicine of Henan Province of China (2019ZY1028).

## Conflict of Interest

The authors declare that the research was conducted in the absence of any commercial or financial relationships that could be construed as a potential conflict of interest.

## Publisher's Note

All claims expressed in this article are solely those of the authors and do not necessarily represent those of their affiliated organizations, or those of the publisher, the editors and the reviewers. Any product that may be evaluated in this article, or claim that may be made by its manufacturer, is not guaranteed or endorsed by the publisher.

## Abbreviations

CLBP: chronic low back pain
LBP: low back pain
BMI: body mass index
NRS: numerical rating scale
RMDQ: Roland-Morris questionnaire
SAS: self-rating anxiety scale
SDS: self-rating depression scale
AUC: area under the curve
ICC: intraclass correlation coefficient
pACC: pregenual anterior cingulate cortex
dACC: dorsal anterior cingulate cortex
fMRI: functional magnetic resonance imaging.
